# Abundance of food leads to triple clutching of Eurasian eagle-owls (*Bubobubo*) and barn owls (*Tytoalba*) in captivity in Bulgaria

**DOI:** 10.3897/BDJ.13.e161110

**Published:** 2025-08-08

**Authors:** Rusko Petrov, Alian Valchev, Hristina Klisurova

**Affiliations:** 1 Green Balkans - Stara Zagora NGO, Stara Zagora, Bulgaria Green Balkans - Stara Zagora NGO Stara Zagora Bulgaria; 2 Trakia University, Stara Zagora, Bulgaria Trakia University Stara Zagora Bulgaria; 3 Vet Centre Stara Zagora, Stara Zagora, Bulgaria Vet Centre Stara Zagora Stara Zagora Bulgaria

**Keywords:** captive breeding, owl conservation, multiple clutching

## Abstract

Food is one of the most important factors for the reproduction capabilities of every living organism. Many studies have highlighted the significance of prey availability on both brood size and offspring survival. In nature, however, it is very challenging to accurately quantify the food intake of the breeding pair. The primary aim of this study is to investigate the reproductive potential of two species in the order Strigiformes, under optimal environmental conditions and adequate nutrition. In some publications, there is evidence for double and even triple clutching. This study evaluates not only the number of clutches, but also the number of eggs laid, fertilised, hatched and released.

## Introduction

Owls are birds from the order Strigiformes and are characterised by their unique futures and mostly nocturnal hunting habits. The order is divided into two main families: Tytonidae - barn owls and Strigidae - typical owls. They include more than 200 species with unique features and behavioural traits distinguishing them from other bird groups (Cholewiak 2003). Even with that huge diversity amongst owl species, there are several key features shared amongst them making their predatory abilities incredible.

One of the most characteristic features of owls, besides their quiet flight, is their facial disc which is an arrangement of facial feathers in a position that aids their sound localisation. The shape of that disc is different in the two families. In strigid owls , such as Eurasian eagle-owl (*Bubobubo*), the disc is symmetrical and round to enhance the bird’s ability to focus sound. On the other hand, the barn owl (*Tytoalba*), from the Tytonidae family, has a heart-shaped disc that enhances the sound detection by funnelling the sound directly to their ears. Owls have asymmetrical ear openings, which allow them to pinpoint the exact location of the sound, even from small prey moving through vegetation and even deep snow. This acute hearing allows them to hunt effectively in complete darkness. Due to their primary nocturnal hunting preferences, their low-light sensory adaptations are crucial. Their eyes are adapted to gathering residual light, allowing them to see in very dim conditions where most other birds would struggle (Scherzinger and Mebs 2024).

Hunting strategies of the owls are remarkable and diverse, shaped by their environment and prey availability. Some owls, like barn owls, are more likely to hunt rodents like Field Vole and Sorex araneus (Facey and Vafidis 2013), while others, like the Eurasian eagle-owl, may hunt larger prey, including larger mammals, amphibians and even other birds (Sándor and Bugariu 2008). Their hunting tactics are adaptable and they can utilise a combination of stealth and power to capture prey, depending on the environment.

In terms of geographic distribution, owls are found in a wide range of habitats. On one side are species like eagle-owls , with their preference for more wild and undisturbed nesting sites, free from human presence. They prefer to settle in areas linked with watercourses, irregular topography and more diverse fauna (Ortego 2007). On the other hand are species like the barn owl adapted more to hunting small mammals in agricultural fields (Milliet et al. 2024). However, other anthropogenic factors such as pollution, habitat destruction and even diseases affecting the prey (Martínez and Calvo 2001) have significant effects on distribution and sustainability, reducing their population in some regions. Most owls are currently classified as “Least Concern” in the IUCN Red List of Threatened Species, yet their population is declining due to many environmental challenges, including habitat loss and climate change (IUCN 2025). One of the key factors is the decline of available catch, which is in direct correlation with the breeding success and offspring survival. There are some studies showing the direct effect between prey population and reproductive productivity of the predator (Pavluvčík et al. 2015). In light of this, conservation strategies must not only focus on maintaining the habitat, but also consider the importance of food availability. Measures to sustain the health of the population of the prey, restoring and maintaining natural hunting grounds and, in some cases, providing supplemental feeding during critical periods like in the breeding season or harsh winters can enhance the stability of the population.

## Material and methods

During 2023 and 2024, one pair of Eurasian eagle-owls and two pairs of barn owls were housed in a controlled environment in the Wildlife Rehabilitation and Breeding Center, part of Green Balkans - Stara Zagora NGO, specifically designed to support their reproductive and behavioural needs. All birds were categorised non-releasable due to behavioural changes resulting from prolonged close human contact or had sustained injuries deeming them incapable of survival in the wild. The Eurasian eagle-owls had been illegally owned and were confiscated. All barn owls were presented with severe wing injuries leading to amputations or at least deformities making them unable to fly. As those injuries are not impeding their parenting abilities, all individuals were initially used as surrogate parents for orphaned and confiscated juveniles. After one year of successful fostering, they were assessed as suitable candidates for captive breeding. The objective was to produce full-fledged generation which eventually can be re-introduced into the wild by hacking (Lazarova et al. 2020).

We used feeding protocols, based on published data of natural dietary composition in the wild population of the species (Saufi et al. 2020, Petrov et al. 2022). Rotational feeding was implemented, providing a variety of protein sources through the week. Rodents were the main part of the diet provided three times a week, rabbits were twice weekly and one-day old chicks or chicken hearts twice. Sundays were designated as fasting days to better simulate natural prey availability cycles. After the first egg hatched, the fasting day was suspended and rodents were given to meet elevated energy demands. For ethical reasons, only freshly euthanised or fresh frozen prey was given. Macro and micronutrients were supplemented with Versele-Laga MutaVit, Versele-Laga Opti-Breed, Versele-Laga Calci-Lux and Masalles Falcon Top. After the start of the breeding season, Masalles Chick Complex and Masalles Probiotic were added. The feeding was done late in the afternoon for better alignment with birds hunting habits (Durant et al. 2013).

## Results

**Barn owls**

**Figure 1.**
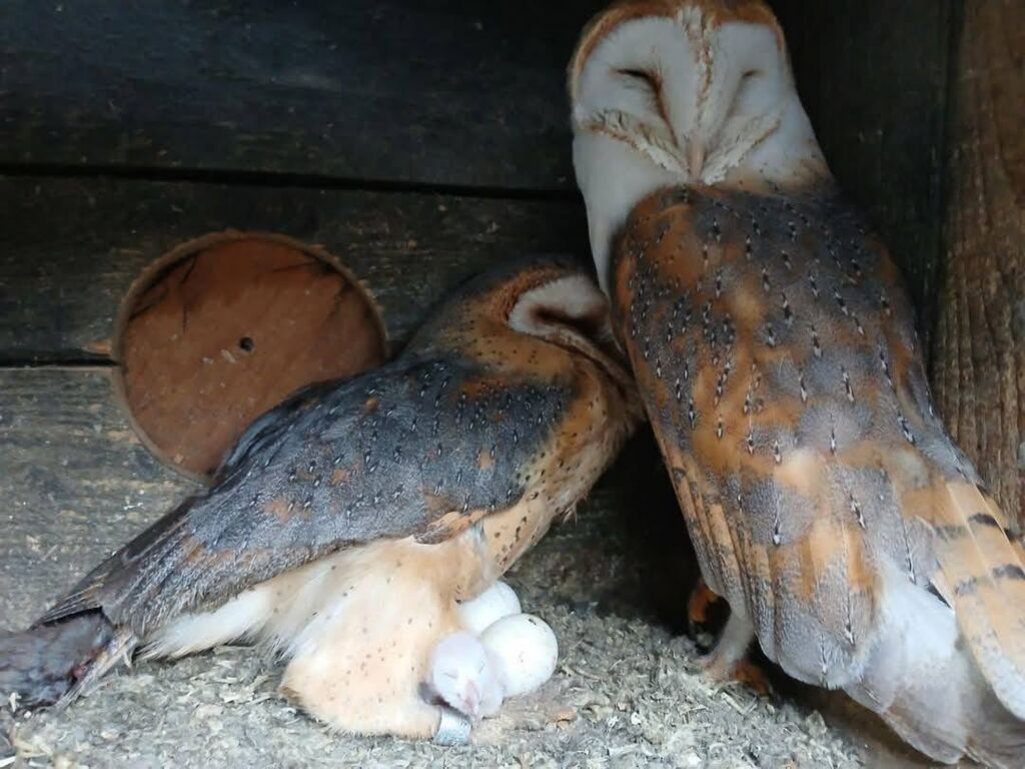
One of the barn owl pairs at the WRBC of Green Balkans.

In 2023, the first pair (Fig. 1) had three clutches of eggs. For the first layng of the year, they produced eight eggs, all fertilised, hatched and released. In the second, they had seven, but one was not fertilised, all six remaining were re-introduced and the third and last produced six viable chicks with no losses. The second pair was more inexperienced, but still had three clutches. On their first attempt for the year, they successfully laid and raised eight eggs. However, in both the second and third attempts, one chick was lost in each, with a total of five youngsters in each clutch. In the second year, the first attempt on both pairs raised eight each with no loss. The second attempt yielded six offspring, a pair with only one unfertilised egg. In the final brooding for the season, the more experienced pair laid and raised five chicks, while the other lost one more, a few days after hatching and nurtured seven. Over the two years of trial, a total of 84 eggs were laid, resulting in the release of 79 birds into the wild (Table 1).

**Eurasian eagle-owl**

The Eurasian eagle-owl produces smaller clutches compared to barn owls. In the first brood for the year, the pair laid and raised two chicks. The second attempt was unproductive, consisting of only one unfertilised egg. The third clutch, produced three weeks later, resulted in two additional chicks with no egg loss. In the second season, the first layng was successful again with two viable offspring. However, both chicks from the second clutch were lost before they could be moved in the hack. On the final attempt, only one juvenile was released from two fertilised eggs. Over the two year study period with a total of eleven eggs laid, seven birds were released into the wild (Table 2).

## Discussion

It is important to acknowledge that this study was conducted on individuals with some physical impairments. Although those limitations only affected their flying and not reproductive and fostering capabilities, they still represent a confounding factor and should be taken into account when interpreting the results. The birds dependency on human-managed care, including food provision and danger free conditions, create an environment more conducive to reproduction than what is typically observed in the wild.

Habitat quality is a well-documented factor for reproductive success. Some studies (Bond et al. 2005) have emphasised the importance of sustainable nesting sites and overall environmental stability in shaping breeding outcomes. On the other hand, data indicate that the disturbance of the nest leading to abandonment of the site and second attempt for nesting did not have a noticeable effect on the quality of the brood, but if it happened again, the third attempt was never done in the same year (Bettega et al. 2011).

Previous publications have reported cases of double (Ortego 2004, Beziers and Roulin 2016) and even triple clutching (Taylor 1994, Scherzinger and Mebs 2024) in barn owls. There is one publication of a possible second brood of eagle-owl (Martínez et al. 2003). However, these observations often lack comprehensive data on egg fertility, hatching success and fledgling survival. The underlying factors contributing to the multiple brooding events remain poor with other factors taking effect like the timing of the first layng (Jackson and Cresswell 2017) and seasonal weather conditions (Charter et al. 2017). Isolating some of those factors can improve the understanding of the events leading to the production of multiple clutches and some benefits of that are already recognised. It is suggested that female barn owls, capable of producing a second brood in a year at least once during their lifetime, can yield more than double the number of fledglings and recruited offspring compared to those that reproduce only once (Zabala et al. 2019).

Another thing that needs to be noted is that all eggs were hatched in the nest and the chicks remained with the parents until they were capable of independent feeding and had left the nest on their own. This approach contrasts with other techniques aimed at increasing reproductive output, in which eggs are removed shortly after layng (Durant et al. 2009). In those methods, the removal of eggs induces the birds to initiate a new clutch mimicking natural scenarios, such as predation or egg loss. In our research, the classification of new clutch is only if the previous cycle is complete and the birds had successfully hatched offspring on their own. Through our study, there were some instances of adults initiating a new clutch even before the chicks from the previous one were released.

The small sample size of the study limits the generalisation of the findings. While the results are encouraging and provide insight of the reproductive potential of the species observed, they are based on optimal conditions and cannot be extrapolated to all individuals and circumstances. However, this study serves as a valuable baseline, demonstrating what may be possible under well-managed conditions and offering a foundation for future, larger scale research.

## Conclusions

This study provides clear evidence that both species of barn owls and eagle-owls are biologically capable of producing three clutches per year when put in optimal conditions. Abundant and species appropriate nutrition combined with good environmental conditions improve parents productivity despite some physical limitations. Although the sample size was limited and involved only injured or human-imprinted individuals, the results highlighted the significant potential of controlled breeding programmes in raptors' conservation. Furthermore, this study reveals that use of non-releasable birds in fostering and breeding roles is a valuable strategy to enhance recovery efforts of the declining owl population.

## Figures and Tables

**Table 1.** The barn owls' breeding output.

First pair barn owls	Laid eggs	Fertilised eggs	Raised eggs	Released eggs	Second pair barn owls	Laid eggs	Fertilised eggs	Raised eggs	Released eggs
First clutch 2023	8	8	8	8	First clutch 2023	8	8	8	8
Second clutch 2023	8	7	7	7	Second clutch 2023	6	6	5	5
Third clutch 2023	6	6	6	6	Third clutch 2023	6	6	5	5
First clutch 2024	8	8	8	8	First clutch 2024	8	8	8	8
Second clutch 2024	7	6	6	6	Second clutch 2024	6	6	6	6
Third clutch 2024	5	5	5	5	Third clutch 2024	8	8	7	7
**Total**	**42**	**40**	**40**	**40**		**42**	**42**	**39**	**39**

**Table 2.** The Eurasian eagle-owls' breeding output.

First year eurasian eagle-owls	Laid eggs	Fertilised eggs	Raised eggs	Released eggs	Second year eurasian eagle-owls	Laid eggs	Fertilised eggs	Raised eggs	Released eggs
First clutch 2023	2	2	2	2	First clutch 2024	2	2	2	2
Second clutch 2023	1	1	0	0	Second clutch 2024	2	2	0	0
Third clutch 2023	2	2	2	2	Third clutch 2024	2	2	1	1
**Total**	**5**	**5**	**4**	**4**		**6**	**6**	**3**	**3**
